# Child physical abuse: changes over ten years in the perceptions of Finnish dental professionals

**DOI:** 10.2340/aos.v83.41058

**Published:** 2024-07-10

**Authors:** Heikki Alapulli, My Blomqvist, Sari Koskinen, Sarimari Tupola, Elisa Valkama, Eeva Nikkola

**Affiliations:** a Children’s Hospital, Department of Oral and Maxillofacial Diseases, Helsinki University and Helsinki University Hospital, Finland; b Department of Oral and Maxillofacial Diseases, Oulu University Hospital, Finland; c Children’s Hospital, Department of Children and Adolescents, Helsinki University and Helsinki University Hospital, Finland

**Keywords:** Child maltreatment, education, mandatory reporting, dentist, dental nurse, physical abuse

## Abstract

**Objective:**

This study aimed to assess perceptions and actions taken by Finnish dental professionals in suspected cases of child physical abuse (CPA) and to describe changes over 10 years.

**Material and methods:**

Data collected from two child abuse and neglect (CAN) surveys among Finnish dental professionals, working in public health care, covering suspicions of CPA and actions taken as well as training on CPA issues, were compared. The chi-squared (χ^2^) test was used to analyze associations.

**Results:**

In total, 625 (2008) and 1,025 (2019) questionnaires were completed. Respondents reported that they suspected CPA more frequently in 2008 than in 2019 (21.0% vs. 8.7%, *p* < 0.001). Out of all respondents, 1.1% had reported their concern to the police in 2019. Worries about the report’s negative consequences to the child at home (44.5% vs. 56.4%, *p* < 0.001) and to the informer (30.2% vs. 36.3%, *p* = 0.016) increased between the surveys. The proportion of respondents with previous training on CPA issues increased between the surveys (5.9% vs. 36.4%, *p* < 0.001).

**Conclusions:**

Recognition of CPA was low and decreased over the years. Furthermore, mandatory reporting to the police was low. Additional education on issues related to CPA is needed.

## Introduction

Violence against children is a common phenomenon globally. It is estimated that worldwide up to one billion children, aged 2–17 years, have experienced physical, sexual, or emotional violence or neglect in the past year [1]. Child physical abuse (CPA) refers to an intentional act in which someone purposefully hurts or harms a child or young person [2]. A recent nationwide Finnish study revealed that 14% of 4-year-olds had experienced physical abuse [3]. Experience of physical abuse is also quite common among schoolchildren in Finland. The latest Finnish School Health Promotion Study in 2023 revealed that 19% of boys and 12% of girls in grades 4–5 have experienced physical threat at least once during the previous year. The figures in grades 8–9 were 21% of boys and 15% of girls, respectively. In grades 4–5, physical abuse from their parent or legal guardian was experienced by 18% of boys and 14% of girls. The same figures in grades 8–9 were 9% of boys and 16% of girls [4].

Child physical abuse, like any other form of child maltreatment, is detrimental to the child’s development and wellbeing [5]. Identification of CPA without delay is essential to prevent further abuse and in the worst situation, the death of the child [6, 7]. Injuries to the head, face, mouth, and neck are not uncommon among physically abused children, although we still lack good quality scientific evidence of pathognomonic clinical findings in oro-facial region [8, 9]. Therefore, all dental professionals are in a unique position when it comes to recognizing possible CPA. Nevertheless, it has been observed earlier that dental professionals could be more active in reporting their suspicion of possible CPA [10–12].

Many countries have legislation to safeguard the child’s safe environment for growth. Finland banned all physical punishment of children in 1984, the second nation in the world to do so [13]. Since then, all health care personnel have been obliged to refer their concern for a child or adolescent in need of additional social support to Child Welfare Services (CWS). The Finnish Child Welfare Act was revised in 2012, whereafter all health care personnel working with children are obligated to report their suspicion of sexual abuse both to CWS and to the police. Moreover, this alteration was expanded to cases of suspicion of CPA and severe child neglect in 2015 [14]. In Finland, all people working with children have a legal obligation to report their suspicion of child maltreatment to the proper authorities immediately and without confidentiality provisions.

Dental care, free of charge, is offered to all Finnish children and adolescents up to the age of 18 in public dental healthcare clinics [15]. Oral health examinations should be offered when the child is approximately 1, 3, 5, 7, 11 and 15 years old. Under a 2011 Government Decree, oral health examinations of children can be conducted by a dental nurse, a dental hygienist, or a dentist [16]. After this Government Decree, dental hygienists and dental nurses see child patients far more often than dentists do in Finland.

In our previous study, we showed that Finnish dental professionals do not recognize and far too seldom report possible child abuse to CWS, even though it is a statutory obligation [17]. Several studies from other countries have established a varying level of knowledge and further need for education on this topic among dental professionals [18–25]. There are only a few Finnish studies of the ability of health care personnel to identify possible CPA [26, 27] and no previous studies of dental professionals.

The aim of this study was to assess the perceptions and actions of Finnish dental professionals regarding CPA and compare the results with the unpublished data from the year 2008. We were especially interested in the effects of the amendment of the law in 2015 on the answers.

## Materials and methods

### Questionnaires

The questionnaire used in 2008 included seventeen questions concerning the respondents’ gender, age, graduation year, profession, the province where they were working, their knowledge and procedures upon suspicion of CPA, and the need for educational training. The questionnaire was first piloted on a group of dentists and then converted into an online survey. The data was gathered with the Webropol survey tool.

In 2019, a Finnish version of an originally Scottish questionnaire on child protection and dental practitioners designed by Cairns et al. in 2005 [28] was used. We carried out a modified cross-cultural adaptation process to the questions [29]. The original questionnaire was first translated into Finnish by a certificated translator, then adapted to conditions and terminology used in Finland, and after that piloted on a small group of dentists, dental hygienists, pediatricians, and a social worker working in the Children’s Hospital in Helsinki, Finland. After this pilot sample, a few adjustments were made to the questionnaire to improve its understandability. Finally, the questionnaire was translated back into English by the certificated translator and compared with the original and found to be similar to it. The validity and reliability of the adapted questions were not assessed. The questionnaire included thirty-three questions concerning child abuse and neglect (CAN). Seven of them were the same as in the survey conducted in 2008 (Supplement 1). Those seven questions dealt with suspicion of CPA, action taken, fears of reporting and educational training on recognition of CPA. The questionnaire was converted into an online survey (Webropol survey and reporting tool version 3.0, Webropol Inc., Helsinki, Finland).

### Study populations

In the 2008 survey, the web link was sent to all chief dental officers (*n* = 238) working in the public dental service in Finland. They forwarded the link to their dental teams and then emailed back the number of the dental professionals in their teams to the investigator. A follow-up reminder was sent 2 weeks after the first email to the chief dental officers.

In 2019, the web link to the online survey was sent via email to 4,062 active members of the Finnish Dental Society Apollonia and to 4,438 active members of the Finnish Federation of Oral Healthcare Professionals with the permission of these organizations. The members of the two associations represent most of the dental professionals working in Finland. Nowadays, dental nurses undergo a 3-year vocational training program in Finland. They typically work collaboratively with dentists as part of a team. However, those who wish to independently examine children’s oral health must complete in-service training in pediatric dentistry. On the other hand, dental hygienists receive 3.5 years of training at the universities of applied sciences and are equipped to work independently, including with children. Only members who were currently working were contacted. Follow-up reminders were sent 2 weeks after the original survey was distributed. The researchers did not receive access to the email address distribution lists and, when participating in the survey, the anonymity of the respondents (IP addresses) was secured through the survey software. In this paper we included only those respondents who worked in the public dental service because we wanted to compare the results to the 2008 survey and also because the majority of dental care for children in Finland is provided by the public health care system.

### Statistical analysis

The data were analyzed using SPSS version 25 (SPSS Inc., Chicago, IL, USA). The chi-square (χ^2^) test was used to analyze associations between variables. The level of significance was set to 5% (*p* = 0.05). The risk of an event occurring was measured by calculating the risk ratios. The strength of association was tested with Cramer’s *V*-test.

Based on the collected data, we defined three background variables for respondents: (1) dental profession, (2) year of graduation, and (3) municipal population size.

Dental profession was dichotomized into two groups given the unequal distribution of respondents in the original four groups. Thus, the group ‘dental nurses’ consisted of both dental hygienists and dental nurses and the group ‘dentists’ included both general and specialized dentists. The size of the town where the respondent worked consisted of three possible categories: <20,000 inhabitants, 20,000–100,000 inhabitants and >100,000 inhabitants.

## Results

### Characteristics of the respondents

In the 2008 survey, 97 (40.8%) of the 238 chief dental officers working in the public dental service forwarded the questionnaire to their dental team. Out of the 1,929 respondents who received the link, 625 accessed the survey, and the response rate was 32.4%.

In the 2019 survey, 1,586 questionnaires were completed with valid data. Out of the 4,438 dental nurses and dental hygienists, 609 (13.7%) completed the questionnaire, and out of the 4,062 dentists, 977 (24.1%) completed the questionnaire. Of these, 1,025 (64.6%) worked in the public dental service and were included in this study. The total response rate was 18.7%.

Table 1 shows the distribution of gender, age, and dental profession in both surveys. The respondents of the two surveys differed from each other in terms of gender and age. The strength of the association between the age group and survey year was calculated (Cramer’s *V* = 0.282, df = 4, *p* < 0.001). In the 2019 survey, the proportion of respondents who were over 50 years old was greater in the dental nurse group (52.4% vs. 34.3%, *p* < 0.001), but not in the dentist group (48.6% vs. 48.0%, *p* = 0.766).

**Table 1:** Distribution of the respondents in the 2008 and 2019 surveys according to gender, age and dental profession.

Background information	2008, *n* (%)	2019, *n* (%)	*p*
Gender (*n* = 625, *n* = 1,013^a^)			
Female	525 (84.0)	929 (91.7)	
Male	100 (16.0)	84 (8.3)	< 0.001
Age (*n* = 625, *n* = 1,023^b^)			
< 30 years	35 (5.6)	124 (12.1)	
30–39 years	92 (14.7)	233 (22.8)	
40–49 years	236 (37.8)	176 (17.2)	
50–59 years	224 (35.8)	321 (31.4)	
> 60 years	38 (6.1)	169 (16.5)	< 0.001
Dental profession (*n* = 625, *n* = 1,025)			
Dental nurses	277 (44.3)	439 (42.8)	
Dentists	348 (55.7)	586 (57.2)	0.553

a 12 of the respondents did not answer this question.

b Two of the respondents did not answer this question.

### Suspicion of child physical abuse and reporting to authorities

Finnish dental professionals reported encountering possible victims of CPA more frequently in 2008 than in 2019 (21.0% vs. 8.7%, *p* < 0.001). The trend was similar both in the dentist group (26.2% vs. 8.9%, *p* < 0.001) and the dental nurse group (14.5% vs. 8.5%, *p* = 0.012).

In the 2019 survey, 52 respondents in the dentist group (8.9%) and 37 respondents in the dental nurse group (8.5%) reported that they had encountered a physically abused child during their working life. Only eleven (1.1%) of all respondents had reported their suspicion to the police. Of all those who have suspected CPA, 12.4% had reported their concern to the police. The median number of reports was one (Interquartile range 1–3) in the 5-year period. The respondents’ dental profession, year of graduation or size of town where they worked had no effect on the number of reports. Those who had received some training on identifying CPA made reports to the police 3.1 times more often (95% CI: 0.91–10.42, *p* = 0.072) compared to those who did not have any training.

Table 2 shows that, 515 (50.3%) of the respondents have suspected CAN, and 275 (26.9%) of all respondents had made a referral to CWS in the 2019 survey. The self-reported number of non-reporters to the CWS or to the police was 327 (32.1%). All those eleven who had reported their suspicion of possible CPA to the police had also suspected CAN at least once, and four of them (36.4%) admit having suspected, but failed to report.

**Table 2:** The comparison of perceptions and actions taken between individuals who reported their suspicions of child physical abuse to the police and those who did not.

Respondents' actions	Have you ever made a report to the police when suspecting CPA?			
	Yes	No	Total	*p*
Have you ever suspected CAN?^a^				
Yes	11 (100)	504 (49.8)	515 (50.3)	< 0.001
No	0 (0)	508 (50.2)	508 (49.7)	
	11 (100)	1,012 (100)	1,023 (100)	
Have you ever made a report to the CWS?^b^				
Yes	8 (80.0)	267 (26.4)	275 (26.9)	< 0.001
No	2 (20.0)	744 (73.6)	746 (73.1)	
	10 (100)	1,011 (100)	1,021(100)	
Have you ever suspected CAN or CPA, but not reported to the CWS or the police?^c^				
Yes	4 (36.4)	323 (32.0)	327 (32.1)	0.752
No	7 (63.6)	685 (68.0)	692 (67.9)	
	11 (100)	1,008 (100)	1,019 (100)	

CPA: child physical abuse; CAN: child abuse and neglect; CWS: child welfare services.

The numbers of respondents who did not answer the question were:

a two (*n* = 2),

b four (*n* = 4) and

c six (*n* = 6).

**Figure 1:**
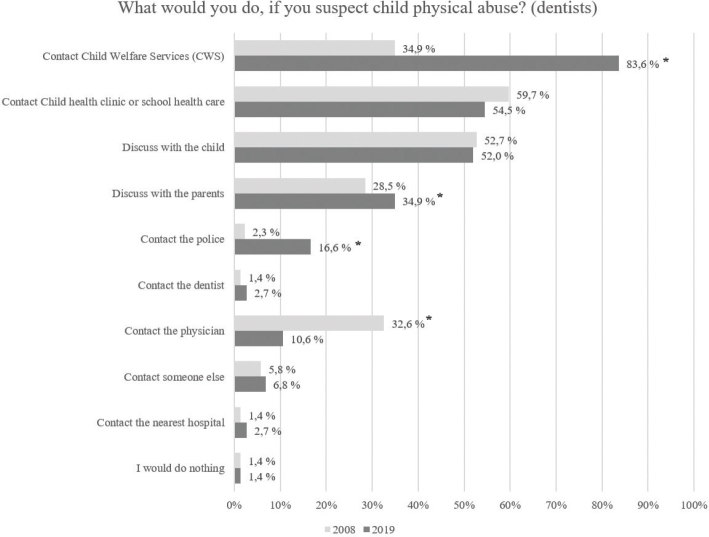
Responses in two surveys regarding dentists’ actions if they suspected child physical abuse (more than one answer was possible).

**Figure 2:**
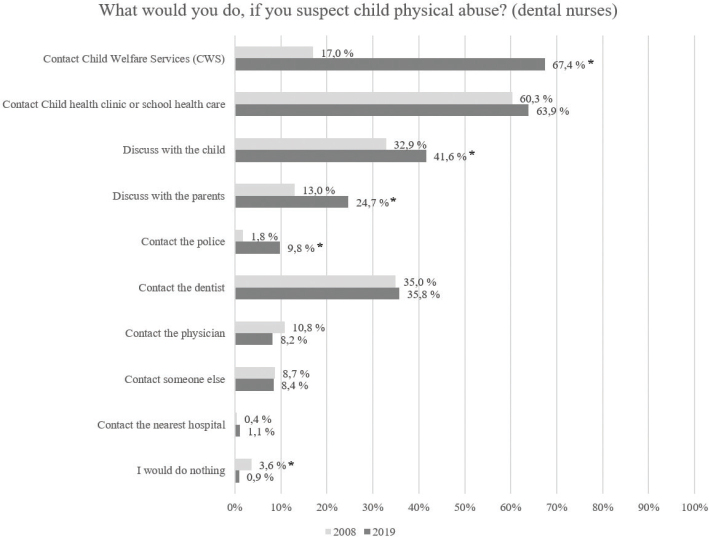
Responses in two surveys regarding dental nurses’ actions if they suspected child physical abuse (more than one answer was possible).

Figure 1 shows the possible actions of the dentists if they suspected CPA and the differences between the answers in years 2008 and 2019. Figure 2 illustrates the corresponding actions of the dental nurses. In the answers of the dentists, there was a statistically significant increase between the surveys for contacting CWS or the police and discussing with parents, and a statistically significant decrease for contacting the physician. In the answers of the dental nurses, there was a significant increase between the two surveys in those who would contact CWS or the police, or discuss with the child or the parents. Correspondingly, the number of those who would do nothing decreased.

### Worries concerning reporting

Table 3 shows the concerns about the consequences of reporting of the respondents according to their profession and the year of the survey. The number of respondents who were worried about the negative consequences to the child at home was smaller in the 2008 (44.5%) than in the 2019 survey (56.4%, *p* < 0.001) and lower in the dentist group (53.1%) compared to dental nurse group (60.8%, *p* = 0.014). The number of respondents who were worried about getting in trouble themselves increased between the two surveys (30.2% vs. 36.3%, *p* = 0.016), and the dentists were more concerned about this in the 2019 survey (38.8% vs. 32.7%, *p* = 0.047). The training received by respondents on CPA issues did not show any association with the questions related to worries.

**Table 3:** The concerns of respondents about the possible consequences of their reports of child physical abuse according to profession and survey year.

Dentists	2008 (*n* = 348) *n* (%)	2019 (*n* = 586) *n* (%)	*p*	Dental nurses	2008 (*n* = 277) *n* (%)	2019 (*n* = 439) *n* (%)	*p*
Are you worried that reporting a child being physically abused might worsen the child’s situation at home?							
yes	142 (44.9)^a^	309 (53.1)^b^	0.020	yes	108 (43.9)^e^	265 (60.8)^f^	< 0.001
no	174 (55.1)	273 (46.9)		no	138 (56.1)	171 (39.2)	
Are you afraid you might get in trouble if you report a child being physically abused?							
yes	101 (31.5)^c^	226 (38.8)^d^	0.029	yes	71 (28.6)^g^	143 (32.7)^h^	0.267
no	220 (68.5)	357 (61.2)		no	177 (71.4)	294 (67.3)	

The numbers of respondents who did not answer the question were:

a thirty-two (*n* = 32),

b four (*n* = 4),

c twenty-seven (*n* = 27),

d three (*n* = 3),

e thirty-one (*n* = 31),

f three (*n* = 3),

g twenty-nine (*n* = 29) and

h two (*n* = 2).

### Training on identification of child physical abuse

The proportion of dental professionals who had had training on identification of physical child abuse increased between 2008 and 2019 (5.9% vs. 36.4%, *p* < 0.001). As seen in Table 4, the dentist group had received more training in both surveys compared to the dental nurse group, but the difference was statistically significant only in the 2019 survey (48.0% vs. 20.8%, *p* < 0.001). The majority of both professions in both surveys reported a desire for further training on identification of CPA, but this desire was less common in the dentist group (83.0% vs. 89.5%, *p* = 0.022 and 89.6% vs. 96.3%, *p* < 0.001). In all, the desire to participate in further training on CPA issues increased between the years 2008 and 2019 (85.9% vs. 92.5%, *p* < 0.001).

**Table 4:** Dental professionals’ training on identification of child physical abuse and their desire to have further training, according to the year of the survey.

Dentists	2008 (*n* = 348) *n* (%)	2019 (*n* = 586) *n* (%)	*p*	Dental nurses	2008 (*n* = 277) *n* (%)	2019 (*n* = 439) *n* (%)	*p*
Have you received training on detecting child physical abuse?							
yes	26 (7.5)^a^	280 (48.0)^b^	< 0.001	yes	11 (4.0)^e^	91 (20.8)^f^	< 0.001
no	321 (92.5)	303 (52.0)		no	266 (96.0)	346 (79.2)	
Would you like to have further training?							
yes	284 (83.0)^c^	523 (89.6)^d^	0.004	yes	247 (89.5)^g^	422 (96.3)^h^	< 0.001
no	58 (17.0)	61 (10.4)		no	29 (10.5)	16 (3.7)	

The numbers of respondents who did not answer the question were:

a one (*n* = 1),

b three (*n* = 3),

c six (*n* = 6),

d two (*n* = 2),

e zero (*n* = 0),

f two (*n* = 2),

g one (*n* = 1) and

h one (*n* = 1).

## Discussion

The present study is the first study to evaluate Finnish dental professionals’ perceptions and actions regarding CPA and to describe the changes over a 10-year period.

Corporal punishment is a common form of physical violence against children worldwide [30]. Most member states of the Council of Europe (34/47 countries) outlaw corporal punishment of children in all circumstances [30]. However, in many countries, physical punishment of children is still permitted, and the attitude toward corporal punishment and other violence against children is varying across the world [30]. In some countries, reporting of suspicion of CPA is mandatory, and in others, it is not legally required. There is also a variation among countries on the course of action, to whom and how health care personnel should report their concerns [31–34]. Most of the published studies of dental professionals report not only CPA but also CAN issues in the same publication. Therefore, it is challenging to compare studies reliably with one another.

One of our main findings was that dental professionals in Finland reported encountering possible victims of CPA in their working life more frequently in 2008 than in 2019 (21.0% vs. 8.7%, *p* < 0.001). There is no evidence of such a decline in the prevalence of CPA in Finland during this time period. According to the statistical reports of the Finnish Institute for Health and Welfare, the proportion of schoolchildren who reported having experienced physical threats declined slightly from 20.7% (2008) to 17.0% (2019) [35]. The same slight decline in experiences of violence among children and young people is also seen in the Finnish national Child Victim Survey [36].

According to our 2019 survey, the frequency of suspicion of CAN was 50.3% (Table 2), which is high compared to those who reported encountering a possible victim of CPA (8.7%). CAN is a wider definition which includes many types of child maltreatment, including physical abuse. One explanation for the difference in suspicion of CPA between the years could be explained by the consolidation of the use of Finnish-language terminology over this 10-year period. When the first survey was conducted in 2008 in Finland, the terminology used in cases of suspected child maltreatment was quite new for dental professionals, and the answers concerning CPA could also include other forms of child maltreatment rather than CPA alone.

Our finding in the 2019 survey, where only 9.0% of the dentists and 8.5% of the dental nurses had suspected CPA in their working life, differs from a Brazilian study published in 2021[37], where 40.3% of the dentists had suspected CPA. Another Brazilian study among primary care health professionals revealed that 57.3% of the respondents had recognized a possible CPA case, and of these, 41.3% had reported their suspicion to the authorities [38]. Our findings concerning suspicion of CPA in both studies (conducted in 2008 and 2019) are low compared to these Brazilian studies. The incidence of CPA in Finland is lower compared to Brazil, and the nature of violence against children is different in these countries [36, 39, 40]. However, Brazilian legislation is comparable to Finnish legislation, as in both countries it is mandatory to report possible CPA cases, and both also prohibit corporal punishment [30, 41].

Between the two surveys conducted in Finland, there have been significant legislative changes concerning professionals’ obligation to report suspected CPA. The Finnish Child Welfare Act was revised in 2015, whereafter all health care personnel working with children were obligated to report their suspicion of physical abuse both to CWS and to the police [14]. Prior to this, the reporting obligation was only to CWS, who in turn reported the suspicion to the police.

Based on our study in 2019, which was conducted after these legislative alterations, out of those who had suspected CPA (*n* = 89), only 12.4% (*n* = 11) had reported their concern to the police (1.1% of the whole study population). For comparison, the proportion of reporting in our investigation was below that observed in a recent Brazilian study, where 6.1% of all respondents had reported their suspicion to the authorities [37]. The frequency of non-reporting behavior (87.6%) in our study is very high compared with earlier studies among health professionals [38, 42, 43].

Reassuringly, our 2019 study revealed that the reporting frequency for suspected CAN was moderate, being higher than that for possible CPA. Specifically, 53.4% of those who suspected any form of CAN made a referral to CWS. Although this is a positive finding, it is important to note that in an ideal situation, the referral frequency for both CPA and CAN should be close to 100%.

The awareness of the amendment is evident in the responses of all dental professionals. In the 2019 survey, the number of professionals who chose the answer options ‘contact CWS’ and ‘contact the police’ to the question ‘What would you do if you suspect child physical abuse?’ increased compared to the 2008 survey. Nevertheless, only 9.8% of the dental nurse group and 16.6% of the dentist group would contact the police, which is alarming and may probably be solved with further training, as reporting to the police is nowadays mandatory. The low real-life frequency of reporting to the police, 1.1% of all respondents and 12.4% of those who had suspected CPA, in the 2019 survey reinforces the impression of the need of continuing education for all dental professionals, as all cases of suspected CPA should be reported without delay to both CWS and the police.

Suspicion of possible CPA may also be emotionally challenging for professionals. Dental professionals are more worried about the child’s situation at home after reporting to the police than before. The same trend is seen in the question concerning the reporter’s own safety. It is possible that dental health care personnel are not as familiar with the police as a cooperation partner as they are with CWS.

On the other hand, awareness of the most serious CPA cases is nowadays widespread because they are reported widely in the media. Sometimes the actions and competence of professionals are also speculated on by the media. This in turn may lead the reporter to be even more conscious of the consequences of their actions when dealing with the CPA cases. The observed increase in respondents’ worries over the years could be partly attributed to this. These worries could even raise the threshold for making the report to the police. Further research is needed to better understand the reasons why dental professionals are currently identifying fewer cases of physical abuse than earlier and why frequency of non-reporting behavior is so high.

Training positively affects professionals’ confidence and knowledge regarding the management of child maltreatment issues [44]. In a repeated cross-sectional study in the UK, there has been a notable upward trend in child protection training over an 11-year period (2005–2016) among pediatric dentists. Additionally, some positive advancements in the suspicion and referral practices over the time were reported [45]. The respondents in our latter survey had received more training on identification of CPA compared to the earlier survey. However, only 48.0% in the dentist group and 20.8% in the dental nurse group had received training on recognizing CPA in the 2019 survey. Particularly concerning is the low proportion of trained dental nurses in the latter survey, since nowadays they are the dental profession that most commonly sees child patients in Finland. These findings support the idea that all dental professionals need more training on recognizing CPA and on their obligation to report, but also concrete advice on what to do in practice. One of the positive findings in our study was that the willingness to attend further training on CPA issues has increased over the years and is high in both profession groups.

One important topic in which dental health care personnel are insufficiently aware is discussion of the suspicion of CPA with the child and the parents. In our 2019 study, 34.9% of dentists and 24.7% of dental nurses responded to the question, ‘What would you do if you suspected CPA?’ that they would discuss their suspicions of CPA with the parents. Health care professionals should not thoroughly discuss the suspicion with the parents nor with the child but leave it to the investigating authorities (i.e. in Finland, the police or a forensic psychologist). Sometimes, especially with small children, a professional’s unintentional leading questions to the child can ruin his/her authentic report, which is important (and sometimes the only) evidence later in court. This can be harmful especially in cases where one or both of the parents are suspected of the CPA.

We should acknowledge some methodological weaknesses in this study. The response rate of our surveys decreased from 32.4% in 2008 to 18.7% in 2019. One reason for this could be the difference in conducting the surveys. In 2008, the survey respondents were invited by chief dental officers, and in the 2019, survey was sent by email. When a supervisor passes on the questionnaire to his or her own dental team, it can have a positive effect on the response rate compared to a standard invitation by email. Compared to earlier CPA studies, both our response rates were modest [10, 46, 47]. There were also differences in the age distribution of the two surveys. In the 2019 survey, the proportion of respondents who were over 50 years old was greater in the dental nurse group compared to 2008 (52.4% vs. 34.3%, *p* < 0.001). In addition, we could have asked if the dental nurses worked independently or as part of a dental team.

In conclusion, there have been some positive alterations in the perception and actions of Finnish dental professionals regarding CPA over this 10-year period. However, it is truly worrying that only a small percentage of respondents followed the Finnish law when suspecting CPA. To address this, Finnish dentists, dental hygienists and dental nurses require additional education on issues related to CPA. In addition to dentists, the competence of dental hygienists and dental nurses is also fundamental given that in Finland these dental professionals see children frequently. Continuing education and training can equip dental professionals with the knowledge and skills to identify cases of CPA, respond appropriately, and most importantly, ensure that the child receives the necessary help.

## Supplementary Material

Child physical abuse: changes over ten years in the perceptions of Finnish dental professionals
