# Supplementary material for: Child physical abuse: changes over ten years in the perceptions of Finnish dental professionals
Source: Acta Odontol Scand. 2024 Jul 10;83:41058. doi: 10.2340/aos.v83.41058 (PMC11302474; doi:10.2340/aos.v83.41058)
Supplement: Child physical abuse: changes over ten years in the perceptions of Finnish dental professionals [file AOS-83-41058-s1.pdf]

Supplementary material has been published as submitted. It has not been copyedited or typeset by Acta Odontologica Scandinavica.

Supplement 1. Questions appearing in both surveys.

1. What would you do If you suspect child physical abuse? (you can choose from several options)
    a. I'll discuss with the child's parent/parents.
    b. I'll discuss with the child
    c. I'll contact the dentist
    d. I'll contact the physician
    e. I'll contact the nearest hospital
    f. I'll contact the police
    g. I'll contact Child Welfare Services (CWS)
    h. I'll contact the Child health clinic or school health care
    i. I would do nothing
    j. I'll contact someone else

2. If you answered the previous section, "I would do nothing", choose what would prevent you from reporting.
    a. Insufficient information
    b. Hurry, there's no time to get down to business
    c. I don't know who to contact
    d. Facing it too hard
    e. I feel I'm violating my confidentiality
    f. Other reason, what

3. Are you worried that reporting a child being physically abused might worsen the child's situation at home? (yes/no)

4. Are you afraid you might get in trouble if you report a child being physically abused? (yes/no)
5. Have you ever met a physically abused child at work? (yes/no)

6. Have you received training on detecting child physical abuse? (yes/no)

7. Would you like to have further training? (yes/no)
